# Supplementary material for: Change characteristics and influencing factors of grassland degradation in adjacent areas of the Qinghai–Tibet Plateau and suggestions for grassland restoration
Source: PeerJ. 2023 Sep 12;11:e16084. doi: 10.7717/peerj.16084 (PMC10503501; doi:10.7717/peerj.16084)
Supplement: Supplemental Information 1 [file peerj-11-16084-s001.docx]

**Calculation of NPP**

ANPP is determined based on the absorbed photosynthetically active radiation (APAR) and light-use efficiency (*ɛ*):

ANPP (*x*, *t*)=APAR(*x*, *t*) × *ɛ*, (1)

where APAR is the incident photosynthetically active radiation (PAR, MJ·m^−2^ ). “*x*” is the spatial location and “*t*” is the time. absorbed by the vegetation per unit time, and “*ɛ”* is the actual light-use efficiency (g·C·MJ^−1^ ). APAR is calculated from the fraction of the total solar radiation (SOL, MJ·m^−2^) accounted for by PAR (FPAR):

APAR (*x*, *t*) = SOL (*x*, *t*) × FPAR (*x*, *t*) × 0.5, (2)

where “0.5” is the proportion of SOL intercepted by the vegetation. Under ideal conditions, the vegetation can achieve its maximum light-use efficiency (*ɛ*_max_), but in reality, this efficiency is constrained by both the temperature and the soil moisture (Xiao et al., 2022) . FPAR can be calculated from the “NDVI”. These constraints are accounted for as follows:

$FPAR_{(x,t)}=\frac{[NDVI_{(x,t)}-NDVI_{i,\min}]-(FPAR_{\max}-FPAR_{\min})}{NDVI_{i,\max}-NDVI_{i,\min}}$, (3)

where NDVI*_i_*_,max_ and NDVI*_i_*_,min_ are the minimum and maximum values of vegetation NDVI, respectively. The values of FPAR_max_ and FPAR_min_ are independent of the vegetation type, and are 0.001 and 0.95, respectively; and “*ɛ*” is accounted for by

*ɛ*(*x*, *t*) = *T_ɛ_*_1_(*x*, *t*) × *T_ɛ_*_2_(*x*, *t*) × *W_ɛ_* × (*x*, *t*) × *ɛ*_max_, (4)

in which “*T_ɛ_*_1_” and “*T_ɛ_*_2_” are the temperature stress coefficients for light-use efficiency, “*W_ɛ_*” is the moisture stress coefficient, and “*ɛ*_max_” is the maximum light-use efficiency under ideal conditions. Yu et al. (2021) used remote sensing, meteorological, and measured “NPP” data to simulate the maximum light energy utilization rate of typical vegetation in China, where grassland is 0.542 g C·MJ^−1^.

The Thornthwaite Memorial model was established based on the data used in the Miami model, but was modified to include Thornthwaite’s potential evaporation model (Lieth et al., 1972). We used the Thornthwaite Memorial model to estimate PNPP (g C m ^−2^ yr ^−1^ ):

$PNPP=3000[1-e^{-0.0009695(\nu-20)}]$, (5) $\nu=\frac{1.05\gamma}{\sqrt{1+(1+\frac{1.05\gamma}{L})^{2}}}$, (6)

*L* = 3000 + 25*t* + 0.05*t*^3^, (7)

In these equations, “*t*” is the time, υ is the annual average actual evaporation volume (mm), “*L*” is the annual average evaporation volume (mm), “*γ*” is the annual total precipitation volume (mm).

HNPP (g C·m^−2^·yr^−1^) is the difference between PNPP and ANPP, and represents the loss or increment of NPP induced by human activities:

HNPP (*x*, *t*) = PNPP (*x*, *t*) - ANPP (*x*, *t*). (8)

Thus, a positive HNPP value represents an NPP loss induced by human activities and a negative value represents an NPP increment produced by human activities.
